# Establishing universal sectioning depth and angle for surgical coronectomy of impacted mandibular third molars: an imaging-based study

**DOI:** 10.3389/froh.2024.1466076

**Published:** 2024-09-19

**Authors:** Kamis Gaballah, Shishir Ram Shetty, Vinayak Kamath, Wael Talaat, Tara Renton

**Affiliations:** ^1^ Department of Oral and Craniofacial Health Sciences College of Dental Medicine, University of Sharjah, Sharjah, United Arab Emirates; ^2^ Department of Public Health Dentistry, Goa Dental College and Hospital, Goa, India; ^3^ Department of Oral Surgery, Faculty of Dentistry, Oral & Craniofacial Sciences, King’s College London, London, United Kingdom

**Keywords:** impacted third molar, coronectomy, inferior alveolar nerve, complications, oral surgery

## Abstract

**Introduction:**

Coronectomy is a safer option than extraction for third molars with an increased risk of injury to the inferior alveolar nerve. However, it can still cause complications due to a lack of standardized and effective tooth sectioning techniques. We proposed a standardized protocol for third molar coronectomy involving standardized tooth sectioning parameters to minimize potential complications, surgical failure, and the need for further procedures.

**Methods:**

The study was conducted on 69 eligible archived CBCTs. The coronal sections of the mandibular at the anterior-most level of the lower third molar were used to determine various axes and reference points. This was done to establish the target angle and depth for the coronectomy sectioning. The data on the depth and angle of the sectioning was presented in means and standard deviation. A multivariate analysis of variance was used to determine the impact of study variables on drill depth and angle. Linear regression and correlation between study variables were also used to predict the drill depth and angle.

**Results:**

The samples included 46 males and 23 females aged from 21 to 47 years. The mean drill angle was determined as 25.01 ± 3.28. The mean drill depth was 9.60 ± 9.90 mm. The bucco-lingual tilt had a significant effect on the drill depth, *F*(1, 62) = 5.15, *p* < 0.05, but no significant impact on the drill angle, *F*(1, 62) = 29.62, *p* > 0.05. The study results suggest that a standardized sectioning protocol can be effective during surgical coronectomy procedures.

**Discussion:**

Drilling at a 25-degree angle to a depth of 9.5 mm is advisable to obtain the desired results. This approach will ensure no remaining enamel is left, minimize the chances of root extrusion and future eruption, and improve the outcome.

## Introduction

Removing symptomatic impacted third molars is the most common surgical procedure in the oral surgery field. There are many indications for the removal of impacted mandibular third molars. Still, the most common cause is a recurrent infection associated with the emerging crown of the tooth, known as pericoronitis. Hence, the primary intent benefit of surgical removal is alleviating the symptoms and signs of pericoronitis and its potential consequences. However, this surgery is associated with a significant rate of complications, including the possible injury of the inferior dental nerve (1–3). Coronectomy is a surgical protocol that removes the dental crown and part of the root but retains the apical part of the root close to the inferior dental canal. It was initially introduced as an alternative to total extraction when the third molar roots are closely related to the inferior dental canal (4). According to the PubMed database, in March 2024, 157 research papers were published on the coronectomy of third molar teeth. Timeline analysis shows that this technique received the most attention between 2018 and 2020, with an average of 19 papers published annually. The most common type of publication was reviews, including systematic reviews, which were the most prevalent at 24 (15.3%) of the total. Clinical studies only accounted for 13 (8%) of the total, leading to only 7 (4.5%) meta-analysis reports focused primarily on reports published between 2002 and 2010. Almost all clinical studies and reviews have shown that coronectomy can be used for treating third molars with high surgical neurologic risks (5–9). Although the surgical technique of coronectomy has been improved over the last two decades, some authors have stressed the importance of further refinements to reduce complications and failure (10, 11). The latter problems are related to the residual part of the roots left *in situ*, resulting from tooth sectioning depth and angle details. Several techniques and proposals have been introduced to mitigate complications arising from drilling during surgical coronectomy of the third molar. These include dynamic image navigation (12) and 3D-printed drilling sleeves (13). These may be utilized during simulation-based training to standardize the technique and increase self-confidence among practicing junior oral surgeons. The clinical use of these techniques was limited to preclinical settings and limited case series reports, often with no control groups. The authors highlighted the problems they faced with the methods, including the need for extensive buccal and distal bone removal. This extended surgical operation time might be associated with more postoperative morbidities and is mainly based on the use of angled highspeed burs rather than standardized straight surgical handpieces. This will likely result in a shallower drilling depth, endangering the root mobility during the coronectomy. Finally, these attempts rely heavily on additional technology, exposing patients to extra radiation, imposing longer waiting times for patients with painful conditions, and incurring additional expenses for both the patient and the healthcare system. Introducing these techniques during simulation-based training can enhance the skills and confidence of junior oral surgeons in performing standardized surgical procedures.

Based on the current literature and the practice pattern, the coronectomy is mainly practiced based on a few papers describing the original technique and its modifications based on the experience of a few expert authors. However, research papers still need to be published explaining how coronectomy sectioning angle and depth can be reproducibly performed to achieve the desired standard outcome. Additionally, all radiological reports discussed earlier required guided surgery, which has proven difficult to accomplish in the current surgical settings. The objective of this study was to establish a standard tooth-cutting angle and depth to achieve the optimal size and position of the remaining root piece. This was carried out by analyzing a large number of impacted mandibular third molars from both genders through radiological observations of archived scans. The goal is to decrease the potential complications that could lead to surgical coronectomy failure and necessitate an additional procedure to remove the retained dental roots of third molars.

## Materials and methods

This investigation was conducted at the College of Dental Medicine, University of Sharjah, in the United Arab Emirates. The research was granted permission by the University Research Ethics Committee (approval number REC-23-09-07-03-F). The study examined 400 Cone Beam CT scans (CBCT) of the mandible. No additional CBCTs were requested for this study. The information gathered was not used to identify the patients. All research data were processed and archived according to the university data protection policies. The CBCT scans were obtained using Galileos CBCT unit (Bensheim, Germany). The scans were acquired using a 15 × 24 cm Field of View (FOV) (voxel size 0.25 mm). The machine was operated at 85 kVp and 7 mA. Assessment of 3D images was carried out using a 1,920 × 1,080 pixel and 23-inch HP monitor screen. A single dental radiologist with ten years’ experience examined the CBCT scans. CBCT scans which included complete crown and root coverage of mandibular third molars were included in the study. CBCT scans with incomplete anatomical coverage of the region of interest (ROI), and scans with missing lower third molars, pathologies or imaging artifacts in the mandibular third molar region were excluded from the study.

**Figure 1:**
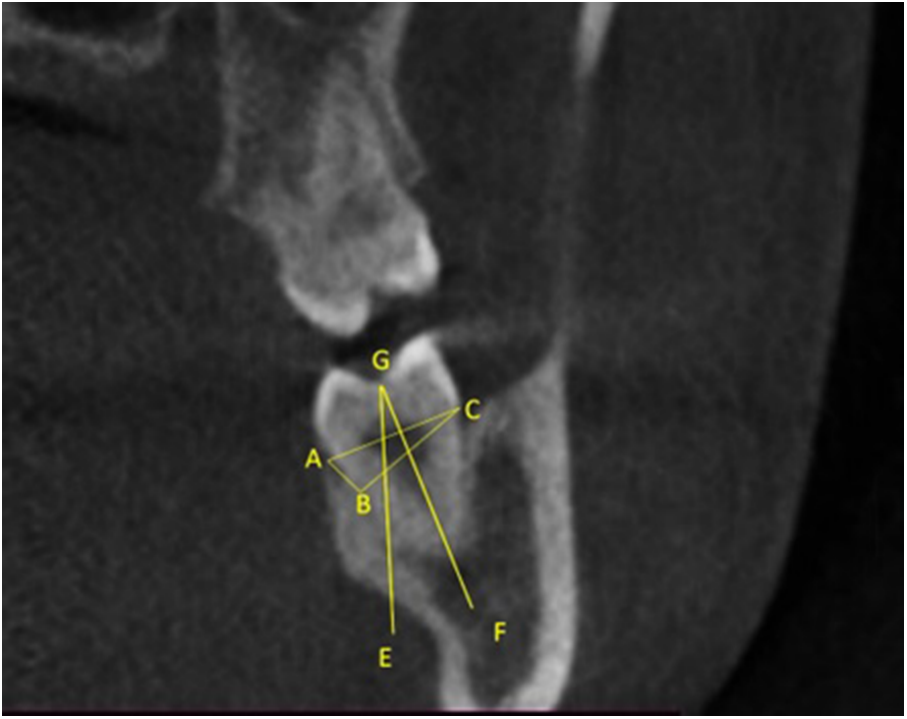
The coronal section of the impacted lower third molar with different axes and angles was used in the study.

The parameters were calculated on the coronal section of the 3D scan. The standard measurement reference point was the anteriormost coronal section, providing full crown and mesial root coverage. A line is drawn from the CEJ on the lingual side of the 3rd molar (point A) extending into point B, which is 4 mm apical to point A along the periodontal ligament space in the Coronal CBCT section. Point B represents the target depth of the coronectomy sectioning to overcome the potential coronal root migration and the emergence of the root through the oral mucosa, i.e., re-eruption. The decision to implement a 4 mm depth for sectioning was based on a comprehensive systematic review and meta-analysis of (13) clinical studies investigating residual root migration. The results of these studies indicated an average root migration of 2.8 mm (6, 7), which led us to conclude that the sectioning depth was the most suitable approach to reduce the chance of eruption of root fragments and the need for subsequent extraction. Point C is marked on the buccal aspect of the 3rd molar at the level of cementoenamel junction such that AC is parallel to the occlusal table. The latter represents the potential entry of the drill for the tooth sectioning from the buccal side. Angle ACB is the target angulation of the drill axis to ensure that the sectioning depth is with target point B and is referred to in this study as the Drill Angle (DA). The distance from point B to C is also measured to determine the Drill Depth (DD). This distance is defined as the optimal depth to ensure almost complete sectioning of the tooth in the Bucco-lingual axis without drilling through the lingual plate of the socket and subsequent damage to the lingual nerve and also to ensure a zero-root movement during the separation of the coronal part of the tooth. To complete the sectioning, the inclined buccal portion of the root fragment must be flattened to the level of point A. The angle EGF formed at the deepest point on the occlusal table by the two lines (mentioned below) is measured to determine the Bucco-Lingual Tilt (BLT); (1) Long axis of the 3rd molar represented by line GF and (2) The vertical perpendicular line drawn from the deepest point on the occlusal surface represented by the line GE (Figure 1).

The data collected was entered into a Microsoft Excel spreadsheet and analyzed using IBM SPSS Statistics, Version 22 (Armonk, NY: IBM Corp). Descriptive data were presented as the mean and standard deviation for continuous variables. Multivariate Analysis of Variance. (MANOVA) was used to assess the influence of study variables on drill depth and angle. Pearson's correlation test was used to test. The correlation between the study variables and linear regression was used to predict the drill depth and angle based on the study variables. A *p*-value less than 0.05 was considered statistically significant.

A pilot study was conducted using 10 CBCT scans that met the eligibility criteria for sample size estimation. Based on a standard deviation of 1.6, a margin of error of 0.5, and an alpha error of 1%, the estimated sample size was determined to be 69 scans.

## Results

All scans were analyzed by a single examiner. The same examiner re-evaluated 10% of the scans from the total analyzed samples after a gap of 15 days. The intra-examiner reliability (intraclass correlation coefficient ICC) was found to be 0.94.

### Study population

An overview of the study population's characteristics is presented in Table 1. Among the 400 CBCT scans 69 scans fulfilled the inclusion criteria. The samples included 46 males (66.7%) and 23 females (33.3%). Participants ranged in age from 21 to 47 years, with the average being 31. The largest age group represented was people under 30, with 30 participants (43.5%), followed by those in their thirties with 29 participants (39.1%).

**Table 1:** Descriptive statistics of drill angle and drill depth based on age and gender.

Age	Gender	N	Drill angle		Drill depth	
			Mean	Std. Dev.	Mean	Std. Dev.
19–30	Male	22	24.26	3.05	9.79	0.95
	Female	8	25.60	3.43	9.41	1.08
	Total	30	24.62	3.15	9.69	0.98
31–40	Male	16	25.44	2.89	9.48	0.76
	Female	11	25.16	4.87	9.39	1.07
	Total	27	25.33	3.73	9.44	0.88
>40	Male	8	25.64	2.92	9.63	0.70
	Female	4	24.63	1.85	10.01	0.85
	Total	12	25.30	2.57	9.76	0.74
Total	Male	46	24.91	2.97	9.65	0.84
	Female	23	25.22	3.88	9.50	1.02
	Total	69	25.01	3.28	9.60	0.90

Std. Dev: Standard Deviation.

### Drill angle and drill depth

The mean drill angle was determined as 24.9 ± 2.97 and 25.2 ± 3.88 for male and female patients, respectively, with an overall mean of 25.01 ± 3.28. The mean drill depth was set at 9.65 ± 8.84 and 9.50 ± 1.02 mm with an overall mean depth of 9.60 ± 9.90 mm.

**Figure 2:**
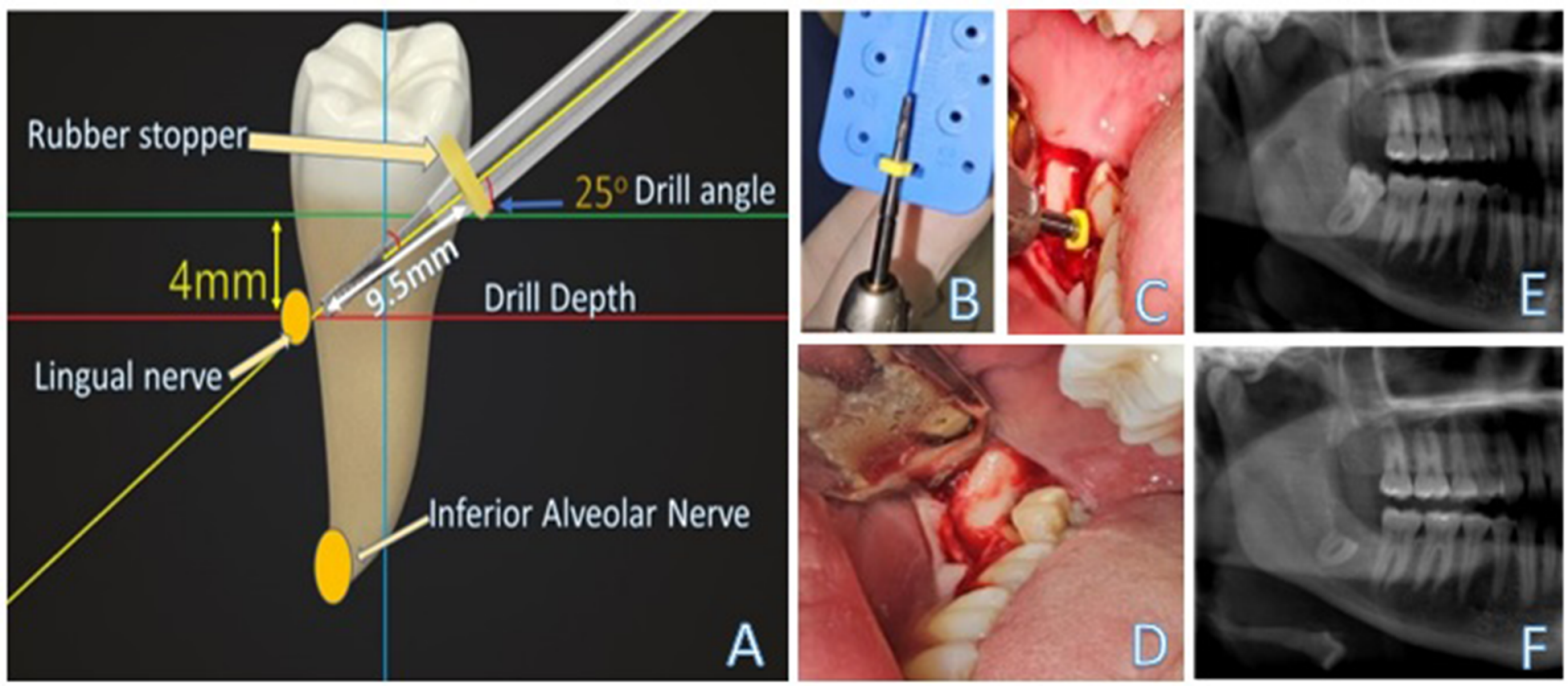
The schematic explanation of the proposed tooth sectioning guide with an example case **(A)** The diagram describes the planning of the tooth sectioning with a drill depth of 9.5 mm at an angle of 25° of the long axis of the tooth to produce complete removal of the crown and leaving the root segment 4 mm inferior to both the buccal and lingual plates; **(B)** Setting up the target drill depth at 9 mm; **(C)** The surgical site was accessed *via* an envelope incision with a short distal incision. The surgical bur was advanced to the predetermined depth at an angle of approx. 25°; **(D)** The sectioning was completed. **(E)** The preoperative panoramic radiograph for the impacted lower third molar with proximity to the inferior alveolar canal; and **(F)** the postoperative radiograph showing no evidence of any residual enamel, clean flat sectioning 4 mm below the crest of the alveolar bone.

Using Pillai's trace, there was no significant effect of age, *V* = 0.04, *F*(4, 124)= 0.61, *p* > 0.05, gender, *V* = 0, *F*(2, 61) = 0.01, *p* > 0.05 and bucco-lingual tilt, *V* = 0.08, *F*(2, 61) = 2.53, *p* > 0.05 on drill angle and drill depth (Table 2). Separate univariate ANOVAs on the outcome variables (drill angle and drill depth) revealed a non-significant effect of age and gender (*p* > 0.05). However, bucco-lingual tilt had a significant impact on the drill depth, *F*(1, 62) = 5.15, *p* < 0.05, but no significant effect on the drill angle, *F*(1, 62) = 29.62, *p* > 0.05. (Table 3). As shown in Table 4, there was a negative weak correlation between drill depth and bucco-lingual tilt (*r* = −0.28, *p* = 0.02). Figure 2 presents a schematic explanation of the proposed tooth sectioning guide with an example case operated based on the outcome of this study.

**Table 2:** The correlation between the drill angle/depth with the study variables: multivariate tests to assess the influence of study variables on drill angle and drill depth.

Effect	Value	*F*	Hypothesis df	Error df	*p*-value
Intercept	0.99	4,358.21	2	61	<0.001*
Bucco-lingual tilt	0.08	2.53	2	61	0.09(NS)
Age	0.04	0.61	4	124	0.66(NS)
Gender	0	0.01	2	61	0.99(NS)
Age* Gender	0.03	0.4	4	124	0.81(NS)

Pillai's Trace, Design: Intercept + Bucco-lingual tilt + Age + Gender + Age* Gender.

* *p* < 0.05 Statistically Significant.

**Table 3:** The correlation between the drill angle/depth with the study variables: linear regression to predict drill angle based on study variables.

	Unstandardized Coefficients		Standardized Coefficients	*t*	*p*-value	95.0% Confidence Interval for B	
	B	Std. Error	Beta			Lower Bound	Upper Bound
(Constant)	49.09	3.57		13.76	<0.001*	41.96	56.22
Age	0.05	0.03	0.15	1.79	0.08(NS)	−0.01	0.11
Gender	−0.06	0.56	−0.01	−0.11	0.91(NS)	−1.18	1.06
Bucco-lingual tilt	0.01	0.03	0.01	0.17	0.87(NS)	−0.06	0.07
Drill depth	−2.70	0.31	−0.74	−8.71	<0.001*	−3.31	−2.08

Dependent Variable: Drill angle, *F* (4,68) = 22.15, *P* < 0.001, R2 = 0.58.

* *p* < 0.05 Statistically Significant.

**Table 4:** The correlation between the drill angle/depth with the study variables: linear regression to predict drill depth based on study variables.

	Unstandardized Coefficients		Standardized Coefficients	*t*	*p*-value	95.0% Confidence Interval for B	
	B	Std. Error	Beta			Lower Bound	Upper Bound
(Constant)	14.70	0.62		23.82	<0.001*	13.47	15.94
Age	0.01	0.01	0.07	0.88	0.38	−0.01	0.02
Gender	−0.08	0.15	−0.04	−0.50	0.62	−0.38	0.23
Bucco-lingual tilt	−0.01	0.01	−0.12	−1.46	0.15	−0.03	0.01
Drill angle	−0.20	0.02	−0.73	−8.71	<0.001*	−0.25	−0.16

Dependent Variable: Drill depth, *F*(4, 68) = 22.44, *P* < 0.001, R2 = 0.58.

* *p* < 0.05 Statistically Significant.

## Discussion

The first technique of coronectomy was proposed four decades ago to reduce the risk of nerve injury in cases of mandibular third molars close to the mandibular canal (4). Since then, many studies and systematic reviews have shown that this technique is safer than complete extraction for treating third molars with an increased risk of IAN injury (6, 9–11). However, coronectomy was not to be a problem-free procedure, and several papers documented the complications of the surgical procedure, including early or late infection, unfavorable root migration, eruption, irritation to the penetrated oral mucosa, and even nerve injuries (14–18). Upon reading these reports, it became apparent that the coronectomy should be refined to reduce the risk of failure. The best approach to such refinement should address the risk factors for the complications reported, and more critically, the procedure should be standardized to produce a consistent outcome when different clinicians perform the surgery. In this study, we attempted to standardize the main component of the coronectomy surgery, the tooth sectioning. When executed well, the latter can minimize the failure and other complications associated with this procedure. We investigated the best drill angle and depth on a good sample size of both genders. The results showed very narrow margins of differences between the optimum drill depth for both gender's teeth. This makes it possible to recommend the average depth of both sexes, around 9.5 mm. This value was related to a target sectioning angle to obtain the required depth of the residual root in relation to the crest of the alveolar bone crest. The best angle to achieve the desired sectioning of the tooth was 25°. Combining the target drill depth and angulation of the drill will prevent retaining residual enamel behind, also known as enamel lipping. The residual enamel was associated with an increased infection rate and coronectomy failure that necessitates the extraction of residual retrieval or at least reoperation to trim the residual enamel portion (19, 20).

An adequate depth could minimize the insufficient drilling before the attempt of crown retrieval, which may trigger the root mobility that necessitates the removal of the root pieces, which means failure of the intended coronectomy and increased risk of nerve injury (20). On the other hand, this could prevent excessive drilling that may lead to the penetration of the lingual plate of the mandible with subsequent mucosal laceration or, to a lesser extent, potential injury to the main trunk of the lingual nerve. Pogrel et al. published two detailed papers that describe the widely practiced coronectomy procedure (21, 22). They suggested the rise of the lingual flap, and the lingual tissues were retracted with an appropriate retractor to protect the nerve. However, it is well known and recently documented in a systematic review and meta-analysis that this practice is likely to increase the lingual nerve injury that is fortunately associated with a temporary altered tongue sensation 2. Hence, we recommend a standard depth and angle that enables the operator to stop the drilling, leaving only 1 mm of dentin to avoid these consequences. The residual 1 mm of the root structure will likely snap easily without micromovement of the root pieces. Our findings indicate that a 2.7-unit increase in drill angle requires a 1-unit advancement in drill depth. This observation has sparked concern regarding using a larger angle, such as 45 degrees, as described by Pogrel et al., (21). Specifically, this approach may significantly increase the depth of tooth sectioning, leading to the possibility of thick residual dentin being left on the lingual side of the tooth. This, in turn, can make the separation of the coronal segment more complex and carry a higher risk of root piece mobility and even extrusion of the root during tooth sectioning. Additionally, the clinician needs to evaluate the buccolingual inclination of the tooth thoroughly. For the teeth with increased buccolingual tilt, there should be a reduction in the depth of the sectioning drill to accommodate that tilt.

Coronectomy can be a practical treatment option for reducing the likelihood of damage to the inferior alveolar nerve in moderate or high-risk cases. However, there are some potential risks associated with this procedure, such as intraoperative difficulties, failure, postoperative infections, root emergence, and the need for further surgery. The authors acknowledge its limitations and await clinical research to verify the proposed protocol's outcome. Nonetheless, this report's critical examination of the angulation and depth of sectioning together in context may help improve the surgeon's understanding of their impact on successful surgical procedures. Adhering to a standardized technique can enhance consistency and improve outcomes, even when coronectomy is performed by clinicians with varying levels of experience.

## Data Availability

The data analyzed in this study is subject to the following licenses/restrictions: dataset can not be shared. Requests to access these datasets should be directed to Shishir Shetty, sshetty@sharjah.ac.ae.
